# Courtship display behavior influences tail myology in *Centrocercus minimus* (Gunnison sage‐grouse)

**DOI:** 10.1111/joa.70089

**Published:** 2025-12-07

**Authors:** Alexander D. Clark, Jessie Atterholt, Samantha J. Clark, Nathan W. Seward

**Affiliations:** ^1^ Committee on Evolutionary Biology University of Chicago Chicago Illinois USA; ^2^ Negaunee Integrative Resource Center, Field Museum of Natural History Chicago Illinois USA; ^3^ College of Osteopathic Medicine of the Pacific Western University of Health Sciences Pomana California USA; ^4^ College of Arts and Science Miami University Oxford Ohio USA; ^5^ Colorado Parks and Wildlife Durango Colorado USA

**Keywords:** endangered, lek, muscle, ornithology, sexual selection

## Abstract

A variety of bird species engage in complicated, elaborate courtship displays to impress potential mates. Such displays include wing flaring, aerial acrobatics, choreographed dances, and tail fanning. Though these behaviors are often well studied, the underlying musculature facilitating them is poorly understood. Exemplars of unique avian courtship behaviors include species of the North American sage‐grouse (*Centrocercus*). Each spring, males gather in leks where they perform courtship displays which incorporate species‐specific sound production, nape feather movement, and importantly, the raising and spreading of their rectricial fan for prolonged periods of time. Here we describe the tail myology of the lekking sage‐grouse species *Ce*. *minimus* (Gunnison sage‐grouse). We compare the tail myology of this species to that of other, either closely related or similarly sized, bird species which do not engage in rectricial displays. Results indicate tail myology in *Ce*. *minimus* is adapted for unique courtship behaviors. Muscles of the rectricial apparatus in *Ce*. *minimus* have greater proportional mass relative to body mass compared to other species examined here, and nearly all other species previously examined. In particular, both overall mass and the mediolateral width of the origination surface of the m. levator caudae are hypertrophied compared to other species which do not incorporate a raised tail fan during courtship displays. Additionally, the muscles that primarily spread the tail fan have relatively more extensive origin surfaces in *Ce*. *minimus*. Our results provide evidence that the specialized courtship behaviors of *Ce*. *minimus* have a clear influence on the tail myology morphology of this species, and suggest that sexually selected displays alter the corresponding underlying musculature across birds.

## Graphical abstract

Courtship displays among birds are widespread, particularly those which incorporate raised tail feathers for extended periods of time. The Gunnison sage‐grouse (*Centrocercus minimus*) is such a species. When we compared the tail muscles of the sage‐grouse to birds which do not engage in erected tail fan postures, both morphology proportional mass significantly differed, particularly in the muscles primarily responsible for raising the rectrices, strongly suggesting courtship display influences myology.

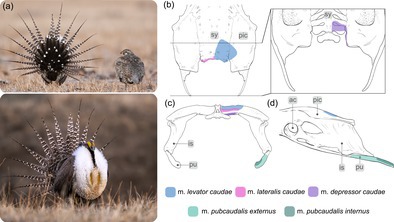

## 1 INTRODUCTION

**FIGURE 1:**
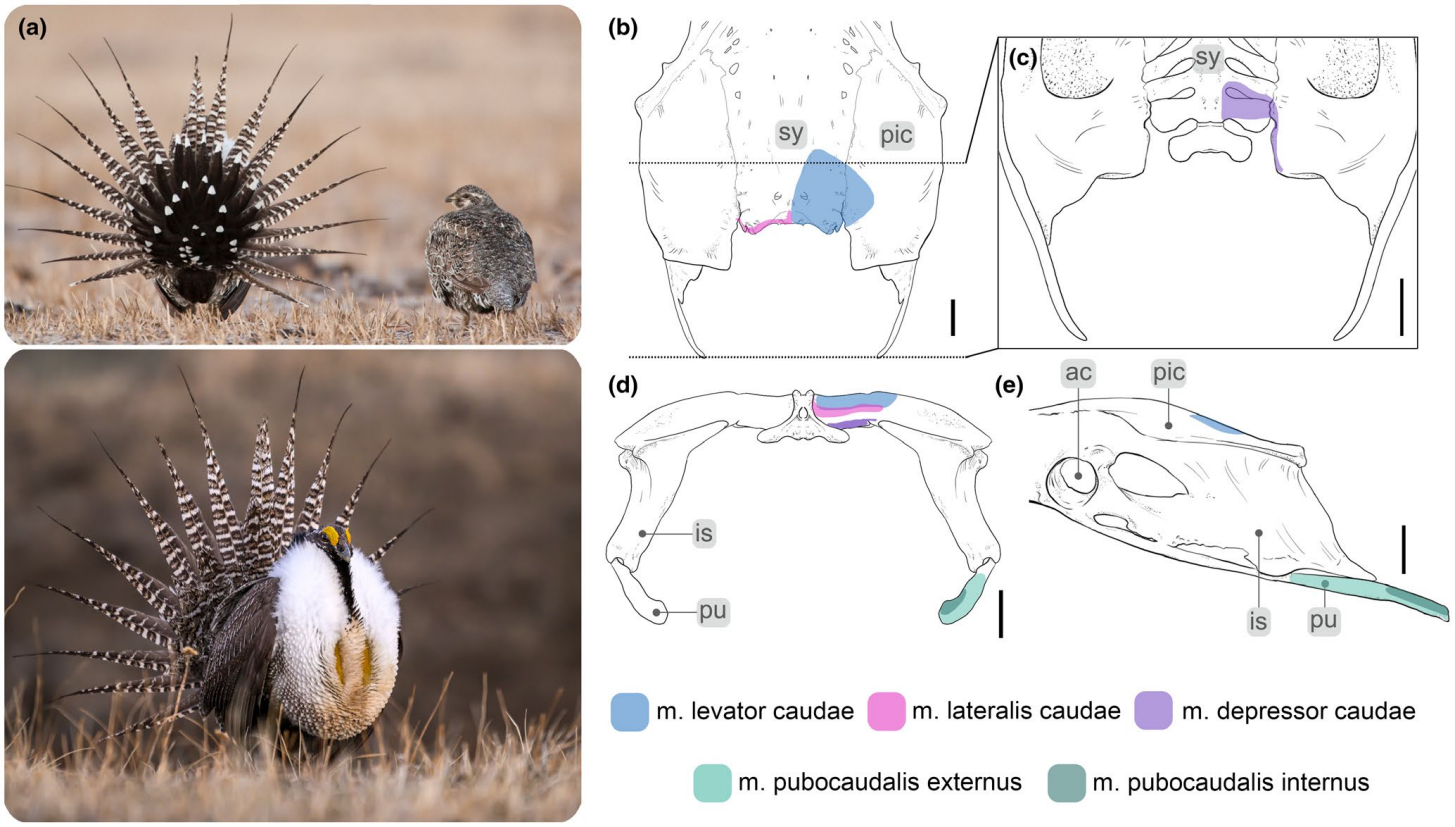
Rectricial muscle origin surfaces of *Centrocercus minimus*. (a) Photos of male *Centrocercus minimus* (Gunnison sage‐grouse) during courtship displays. Photographs by Noppadol Paothong and Seth Owens, respectively, used with permission. Note the fanning and raising of the tail fan during these displays, particularly in comparison to the female. (b‐e) Schematic line drawings of the caudal portion of the pelvis showing origin surfaces of tail muscles. (b) The dorsal view of the pelvis with a (c) ventral view of selected portion also shown, (d) caudal view, and (e) left lateral view. ac, acetabulum; is, ischium; pic, postacetabular iliac crest; pu, pubis; sy, synsacrum. Scale bars each represent 10 mm.

The complex social structures and accompanying feather ornaments of lekking bird species have been of particular interest to evolutionary biologists for decades, typified in genera such as *Paradisaea*, *Tympanuchus*, and *Rupicola* (Cuervo & Møller, 2001; Darwin, 1871; Fitzpatrick, 1998; Frith et al., 1998; Gould & Augustine, 2020; Moynihan, 1963). In these courtship displays, males often gather to form a court (lek) in which they present themselves to on‐looking females to vie for both attention and ultimately the ability to copulate, thus passing their genes to subsequent generations (Lovette et al., 2016). One of the best examples of a lekking species on the North American continent can be found in the genus *Centrocercus* (sage‐grouse) (del Hoyo et al., 1992; Young et al., 2020). In *Centrocercus*, lekking occurs in early spring with individual females only visiting leks for several days out of the entire breeding season (Gibson et al., 1991; Gibson, 1996). Previously considered one species (*Centrocercus urophasianus*), the two—*Ce. urophasianus* (Greater sage‐grouse) and *Ce. minimus* (Gunnison sage‐grouse), represent the largest grouse species in terms of body size in North America (Young et al., 2000). *Centrocercus minimus* is distinguishable from *Ce. urophasianus* in a number of ways including a relatively smaller body size (upper mass thresholds of 2.1 kg compared to 3.9 kg), subtle plumage differences, and disparate lekking dynamic behaviors (Young et al., 1994, 2020). However, a commonality present in the males of both species is a sustained raising and spreading of the rectricial fan, which, when viewed head‐on, forms a near‐circular outline around the body (Figure 1a). This posture is often maintained for hours to successfully attract prospective females and indeed, males of *Ce*. *minimus* which present the most prolonged displays are associated with the greatest mating success: up to 90% of copulations on a given lek attributable to one or two males (Young et al., 1994; Young et al., 2020).

Though the function of tail feather morphology in displays has received much previous attention (Guan et al., 2022; Thomas, 1993; Winquist & Lemon, 1994; Zhou et al., 2023), comparatively little is known about the underlying tail myology. The discussion of how the morphology of these muscles affects function is limited in the literature, and has been previously described in only a handful of avian genera [e.g., *Meleagris* (Galliformes), *Caracara* (Falconiformes), *Tyto* (Strigiformes), *Milvago* (Falconiformes), *Micrastur* (Falconiformes), *Falco* (Falconiformes), *Columba* (Columbiformes)] (Baumel, 1988; Harvey et al., 1969; Lo Coco et al., 2020; Mosto et al., 2018). The primary muscles of the tail region in birds are the mm. levator caudae, lateralis caudae, depressor caudae, pubocaudalis externus, pubocaudalis internus, and caudofemoralis (Baumel, 1988). The combined functions of these rectricial muscles are to raise, lower, pivot, and fan the rectrices, often in flight or when landing (Lovette et al., 2016). During flight, bilateral contraction of the rectricial muscles increases lift by opening the rectricial fan while unilateral contraction aids in steering or pivoting (Gatesy & Dial, 1993; Moreno, 1996). During landing, ventrolateral spreading of the rectricial fan induces drag, and ultimately, stalling to decrease momentum (Gatesy & Dial, 1993; Lovette et al., 2016). These muscles can also be co‐opted for functions other than those strictly related to flight. In many lekking species, like *Ce*. *minimus*, the tail's erect posture is facilitated by the same muscles that produce dorsiflexion of the rectricial fan, albeit to a greater degree and for a longer duration (Young et al., 1994). Though poorly understood, the effects of unique courtship behaviors have led to astounding species‐specific musculoskeletal morphologies (e.g., the skulls of *Cicinnurus* and *Parotia*) (Frith et al., 1998).

This study focuses on the tail myology of a male lekking species, *Ce. minimus*, which uses its tail in courtship displays. The primary aim is to compare the morphology and relative proportions of these muscles with those of non‐lekking birds, in which the tail musculature functions primarily in flight. This comparison provides insights into how modifications of the caudal muscles may be associated with specialized courtship behaviors.

## 2 MATERIALS AND METHODS

### 2.1 Specimen acquisition

A Gunnison sage‐grouse (*Ce. minimus*) (Galliformes, Phasianidae) carcass was collected from within its native range in the Gunnison basin of Southcentral Colorado, USA, in 2024 (Young et al., 2020). The specimen, an individual of an IUCN endangered species, was acquired under Colorado Parks and Wildlife's Agency scientific collection permits #DOW001 and #CPW003 (IUCN, 2025). The individual was found deceased, with the cause of death identified as structural collision. It was collected and placed in a freezer on the day of death. At the time of collection, decay of the leg, neck, and forelimbs muscles had already begun. The individual remained frozen until dissection was conducted, less than 1 year later at the Field Museum of Natural History (FMNH) (Chicago, IL USA). This specimen, now skeletonized, has been integrated into the collections at the FMNH (catalog number FMNH S24‐6833).

The other four bird species were acquired under the FMNH salvage permit #MB675461. Birds were collected either from window strike mortalities, death in captivity, or death during rehabilitation. These individuals were frozen for less than 1 year, and were thawed for dissection and myological study. These species included: *Mergus serrator* (Red‐breasted Merganser) (Anseriformes, Anatidae), *Ardea herodias* (Great Blue Heron) (Pelecaniformes, Ardeidae), *Buteo jamaicensis* (Red‐tailed Hawk) (Accipitriformes, Accipitridae), and *Coturnix coturnix* (Common Quail) (Galliformes, Phasianidae) (Table 2). These taxa were chosen due to their availability for dissection and (with the exception of *Co. coturnix*) approximate body mass similarity to *Ce*. *minimus*. Though much smaller than *Ce. minimus*, assessing the tail myology of *Co. coturnix* allowed for comparisons between two galliforms (Table 2). All individuals were sexually and somatically mature based on body size and plumage.

### 2.2 Muscle dissection and measurement

For each individual, feathers and skin of the pelvic and tail region were removed to fully expose the underlying musculature. The rectricial bulbs and six muscles associated with the tail were described for each species: mm. levator caudae, lateralis caudae, pubocaudalis externus and internus, caudofemoralis, depressor caudae (Table 1). Further analysis focused on the three muscles of the tail that were largest and best preserved in the specimen of *Ce. minimus*, mm. levator caudae, lateralis caudae, and depressor caudae. In this individual, the proximal portions of mm. pubocaudalis externus and internus were too delicate and decayed for this additional analysis, and the caudal portion of the m. caudofemoralis was a very small, minor tail muscle. Using digital calipers (OriginCal1P54), we collected three linear measurements for m. levator caudae, m. lateralis caudae, m. depressor caudae, and for the rectricial bulbs: total proximodistal length, and the mediolateral widths of the proximal‐most and distal‐most margins. The mm. levator caudae, lateralis caudae, and depressor caudae were removed by cutting their bony and soft tissue attachments, and were weighed using an Ohaus navigator gold model scale (0.01 g precision). The muscles on both sides were weighed and averaged, and the percentage relative to body mass was obtained from that average. Body mass ranges of each bird species (accounting for the sex of the individuals) were taken from their respective profiles on the Cornell Lab of Ornithology's birds of the world database (Craik et al., 2020; McGowan et al., 2023; Preston & Beane, 2024; Vennesland & Butler, 2020; Young et al., 2020), and corroborated using sex‐specific ranges in Dunning (2008). Averaged masses were calculated by dividing the upper and lower recorded limits (of the respective sex) by two (Table 2). Muscle and skeletal terminology primarily follow Baumel et al. (1993).

**TABLE 1:** Functional roles of assessed tail muscles. Descriptions of muscle roles within the tail drawn primarily from Baumel (1988), Baumel et al. (1993), and Gatesy and Dial (1993).

	Primary action on the tail
m. levator caudae	Elevates via dorsiflexion of free caudal vertebrae/pygostyle and craniodorsal rotation of rectricial bulbs
m. lateralis caudae	Abduction of rectrices (bilateral contraction); tilt via craniodorsal rotation and elevation of rectricial bulb (unilateral contraction)
m. depressor caudae	Depression via ventriflexion of caudal vertebrae/pygostyle and ventromedial rotation of rectricial bulbs; resists upward displacement during flight
m. pubocaudalis externus	Tilt via depression of rectricial bulb (unilateral contraction); abduction of rectrices (bilateral contraction)
m. pubocaudalis internus	Lateral deviation via lateral flexion of free caudal vertebrae (unilateral contraction)
m. caudofemoralis	Lateral deviation via lateral flexion of free caudal vertebrae (unilateral contraction)

**TABLE 2:** Rectricial muscle metrics. All muscle metrics represent the averaged values between the pair (left and right side of the body). Muscle length denotes proximodistal measurements, whereas width denotes mediolateral measurements. The “length” of the rectricial bulb was measured as lateral extension from the vertebral column. Similarly, proximal and distal width for this structure were taken along the craniocaudal axis.

		m. *levator caudae*	m. *lateralis caudae*	m. *depressor caudae*	Rectricial bulb
	Mass (g)	2.2	0.51	1.2	—
	Percent BM	2.40%	0.55%	1.30%	—
*Ce. minimus*	Length (mm)	56.8	42.4	40.5	32
1.6–2.1 kg	Proximal width (mm)	16.3	4	6.2	10.7
FMNH S24‐6833	Distal width (mm)	8.4	6.9	10.31	12.5
	Mass (g)	0.78	0.29	0.63	—
	Percent BM	1.30%	0.48%	1.10%	—
*Mer. serrator*	Length (mm)	53.04	29	49.4	22.8
0.8–1.35 kg	Proximal width (mm)	5.9	1.4	7	10.2
FMNH S25‐0638	Distal width (mm)	12.3	3.2	7	12.3
	Mass (g)	0.61	0.23	0.66	—
	Percent BM	0.58%	0.22%	0.63%	—
*A. herodias*	Length (mm)	51.7	32.6	23	21.5
1.7–2.5 kg	Proximal width (mm)	7.3	4.3	7.8	8.4
WWH‐16872	Distal width (mm)	5.3	3.4	4.8	10.6
	Mass (g)	1.1	0.31	0.94	—
	Percent BM	1.70%	0.48%	1.40%	—
*B. jamaicensis*	Length (mm)	46.4	45.3	39.5	15.7
1.1–1.5 kg	Proximal width (mm)	10.5	4.8	11.2	10.4
FMNH S25–1119	Distal width (mm)	9	6.3	10	13.4
	Mass (g)	0.064	0.016	0.055	—
	Percent BM	1.20%	0.29%	1%	—
*Co. coturnix*	Length (mm)	26.6	12.5	16.4	4.64
0.07–0.15 kg	Proximal width (mm)	4.7	1.04	4.4	3.64
FMNH S25–0015	Distal width (mm)	1.04	2.54	3.4	3.07

## 3 RESULTS

### 3.1 Tail musculature of *Ce. minimus*

**FIGURE 2:**
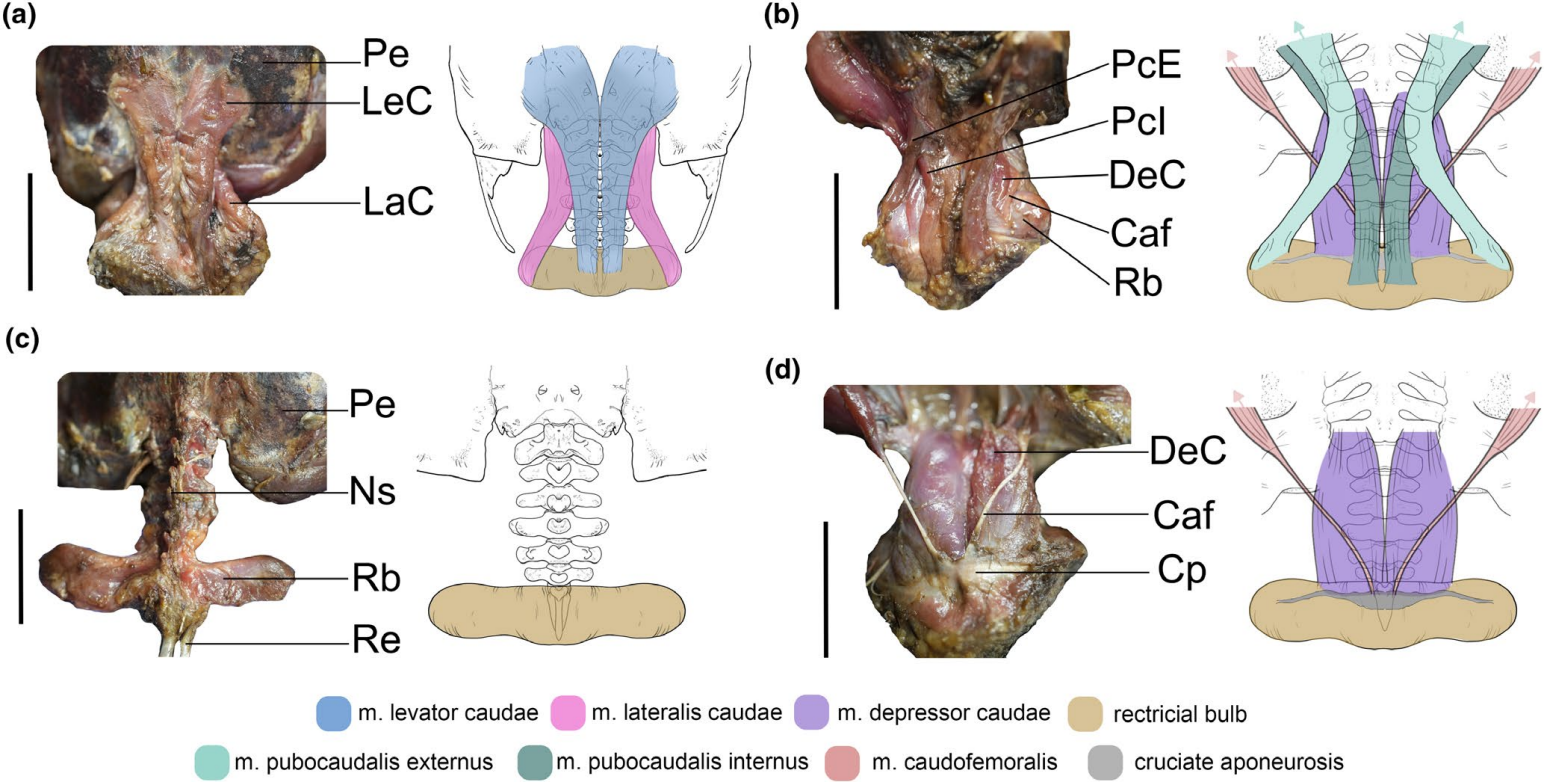
Gross dissection images and schematic line drawings of the tail myology and rectricial bulbs of *Centrocercus minimus*. Each panel shows an image taken during dissection (left) and a line drawing of the same structures (right). (a) superficial dorsal muscles, (b) superficial ventral muscles, (c) rectricial bulbs in dorsal view, and (d) deep ventral muscles. In (b), the. m. pubocaudalis externus was partially decayed and was only visible on the right side, where it was minorly displaced from its natural position; it has been reconstructed bilaterally. In (d), the tendon of m. caudofemoralis was broken on the left side (as seen in the dissection photo) but has been reconstructed as bilaterally intact. Scale bars equal 30 mm and apply to dissection photographs. DeC, m. depressor caudae; Caf, m. caudofemoralis; Cp, cruciate aponeurosis; LaC, m. lateralis caudae; LeC, m. levator caudae; Pe, pelvis; PcE, m. pubocaudalis externus; PcI, m. pubocaudalis internus; Ns, neural spines of the free caudal vertebrae; Re, rectrices; Rb, rectricial bulb.

m. levator caudae. The m. levator caudae has a fleshy origin on the dorsomedial surface of the caudal portion of the synsacrum and postacetabular iliac crests, covering the iliosynsacral suture (Figures 1b,d and 2a). Throughout its length, it also attaches to the transverse processes, laminae, and spinous processes of the free caudal vertebrae via tendinous bands. At the site of origination, the mediolateral width of each muscle measures roughly twice that of the point of insertion, giving the m. levator caudae a well‐developed distally tapering morphology in dorsal aspect (Table 2). It has a tendinous insertion on the dorsal surface of the rectricial bulb in a medial position near the midline.

m. lateralis caudae. The proximal part of the m. lateralis caudae is deep to the m. levator caudae. It has a tendinous origination on the first two free caudal vertebrae via the dorsolateral surfaces of the transverse processes (Figures 1b,d and 2a), as well as an additional unique fleshy origination on the caudal margin of the synsacrum immediately adjacent to the caudal vertebrae, and partially on the medial‐most caudal surface of the postacetabular iliac crest. The muscles insert on the dorsolateral surface of the rectricial bulb as a fleshy sheet. In dorsal aspect, the proximal‐most margin of origination measures approximately 58% that of the distal‐most margin of insertion, thus exhibiting a distally expanded morphology.

mm. pubocaudalis externus and internus. The m. pubocaudalis externus has a fleshy origination on the distal half of the pubis and inserts on the ventrolateral face of the rectricial bulb. Deep to the m. pubocaudalis externus, the m. pubocaudalis internus also has a fleshy origination along the pubic shaft (Figures 1d,e and 2b). In the ventral aspect, the thin, strap‐like muscle bellies of the m. pubocaudalis internus are positioned medially relative to the m. pubocaudalis externus, arranged in parallel near the midline of the tail. The mm. pubocaudalis externus and internus have both fleshy insertions, in lateral and medial positions located on the ventral surface of the rectricial bulb, respectively (Figure 2b).

m. caudofemoralis. The caudal region of m. caudofemoralis is present as a small, elongate muscle emerging from a position deep to m. flexor cruris medialis as it approaches the uropygium. The muscle is distally fusiform in shape, with a thin, long tendon the same length as the muscle belly (Figure 2b,d). The tendons of m. caudofemoralis pass obliquely over the uropygium (superficial to m. depressor caudae), from a lateral position proximally to a medial position distally, where they insert on the rectricial bulb via the cruciate aponeurosis (Baumel, 1988; Figure 2b,d).

m. depressor caudae. The m. depressor caudae has a fleshy origination both within the body cavity on the caudomedial ventral surface of the synsacrum and along the medial part of the synsacrum's thin caudal margin (Figure 1c,d). It inserts on the proximal portion of the rectricial bulb via the cruciate aponeurosis (Baumel, 1988), together with m. caudofemoralis (Figure 2b,d). Along its length it attaches along the ventral faces of the transverse processes of free caudals. The mediolateral width of the proximal‐most margin of origination measures approximately 60% that of the distal‐most margin of insertion, giving each of the muscles a distally expanded morphology. Differentiation of the muscle into mm. depressor caudae profundus and superficialis was not discernible.

### 3.2 Rectricial bulbs of *Ce. minimus*

The paired rectricial bulbs form a horizontal bar, perpendicular to the vertebral column (Figure 2c). Each bulb projects laterally from the pygostyle and has a distally rounded margin. They are wider mediolaterally than craniocaudally. On the ventral face of the rectricial bulbs, the tendinous cruciate aponeurosis acts as a common insertion surface for the m. depressor caudae and the distal tendons of the m. caudofemoralis. The bulbs are located between the mm. levator caudae and the depressor caudae; the proximal parts of the bulb are obscured by these muscles, though the lateral‐most portions are visible without the former muscles being reflected. Each bulb is covered by a thin, sheet‐like m. bulbus rectricium. [Correction added on 23 December 2025 after first online publication: The section heading has been corrected from “Rectricial bulbs are of Ce. minimus” to “Rectricial bulbs of Ce. minimus”.]

### 3.3 Proportional muscle masses across sampled species

**FIGURE 3:**
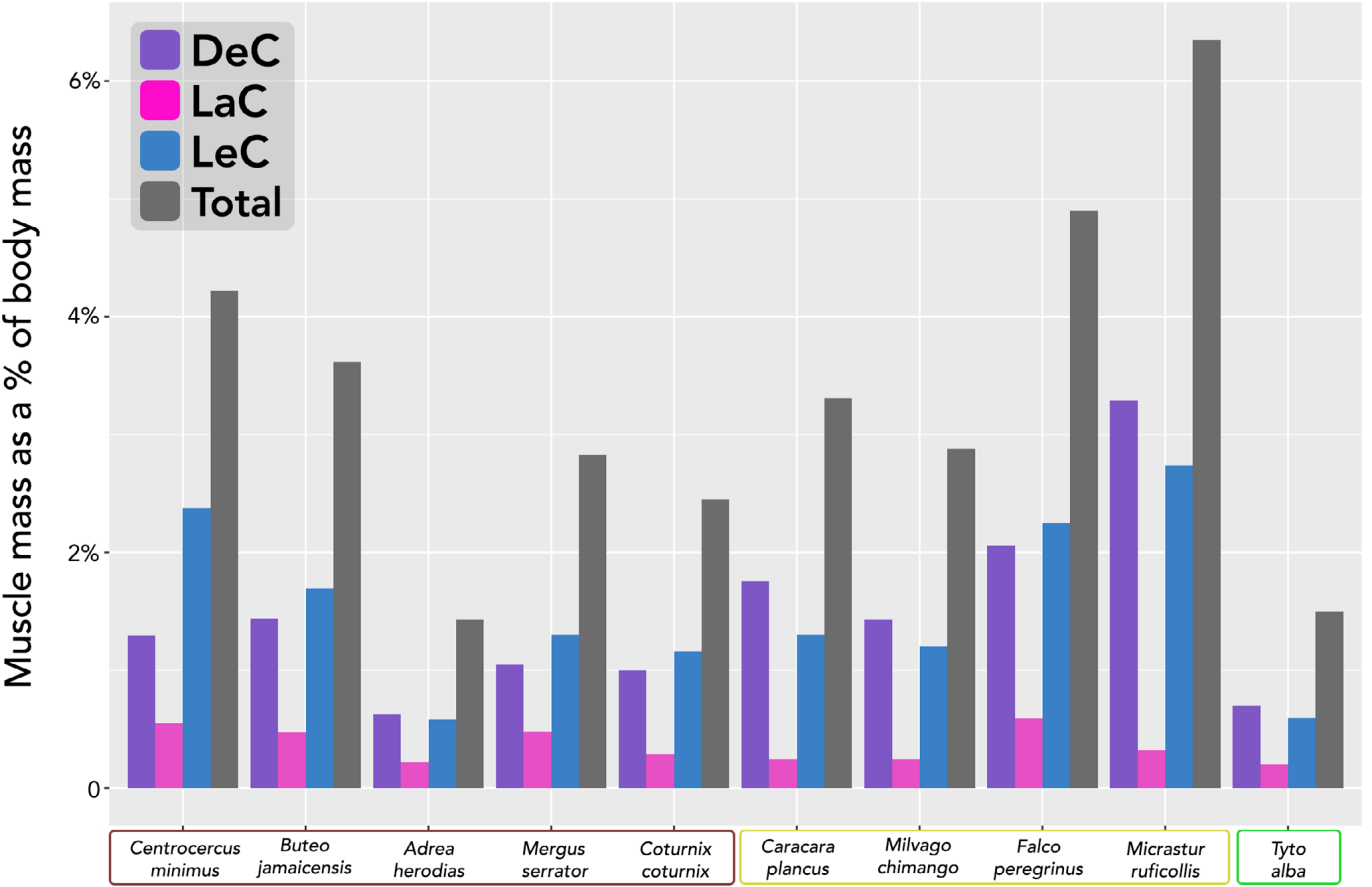
Rectricial muscle masses in proportion to body mass. Masses of the three primary muscles of the rectricial apparatus in species sampled in this study and in species with previously published data. The yellow outline denotes taxa from Mosto et al. (2018), green outline denotes the taxon from Lo Coco et al. (2020), and red outline denotes data gathered for the current study. To note, data from Mosto et al. (2018), removed and weighed muscle pairs separately and summed to achieve a proportional body mass value. Lo Coco et al. (2020) removed and weighed the muscles from one body side, then doubling its value to get achieve a proportional body mass value. Even though proportional muscle masses are higher among some falconids, *Ce*. *minimus* retains the greatest disparity in proportional mass between the m. levator caudae relative to the m. depressor caudae. DeC, m. depressor caudae.; LaC, m. lateralis caudae.; LeC, m. levator caudae.

Among the five species dissected for this study, the m. levator caudae is largest relative to body mass, and in *Ce. minimus*, this is proportionally greater than in other taxa (Table 2; Figure 3). The proportional mass of the m. depressor caudae is greatest in *B. jamaicensis*, followed by *Ce. minimus* and *Mer. serrator*. The proportional mass of the m. lateralis caudae is nearly equal in *Ce. minimus* and *B. jamaicensis*, followed closely by *Mer. serrator*. The total mass of these muscles compared to the total body mass of their respective species is greatest in *Ce*. *minimus* (Figure 3), followed by *B. jamaicensis* Mer. serrator, Co. coturnix, and A. herodias (Table 2; Figure 3).

## 4 DISCUSSION

Results suggest that the muscular morphology of *Ce*. *minimus* differs from other birds which lack courtship displays incorporating sustained raising of the rectrices. Additionally, both the proportional mass of the m. levator caudae and the overall total mass of the caudal muscles relative to body size are greater in *Ce. minimus* than other assessed species here, even when accounting for body size and relatedness. As *Ce*. *minimus* is a ground‐dwelling species considered to be a weak flier, the observed atypical proportions and morphologies of these muscles are likely attributable to their use in courtship displays rather than locomotion (Young et al., 2020). Here we discuss the implications of our findings and how they support the conclusion that unique behaviors are coupled with underlying unique musculature.

### 4.1 Unique dorsiflexion and lateroflexion of the tail facilitated by atypical musculature

One of the most notable findings is the atypical size and morphology of the m. levator caudae in *Ce*. *minimus*. Though it is superficially similar in morphology to other galliforms (*Mel. gallopavo* and *Co. coturnix*) in that it is a flat muscle with a fleshy origin and tendinous insertions that taper distally, this muscle in *Ce*. *minimus* has the greatest mass relative to the other primary muscles of the tail of any species examined here or previously, with the exception of *Micrastur* (Mosto et al., 2018) (Figure 3). However, unlike any other species, the m. levator caudae is nearly double the mass of the m. depressor caudae—the next largest muscle. This is opposite to reported findings of Mosto et al. (2018) for some falconiforms in which the m. depressor caudae is described as the “most‐well‐developed” muscle of the tail. In nearly all other species, including the galliform *Co*. *coturnix*, the total mass of the m. levator caudae is nearly equal to that of the m. depressor caudae (i.e., ratio closer to 1:1) (Lo Coco et al., 2020; Mosto et al., 2018) (Table 2, Figure 3, Table S1). The m. levator caudae in *Ce. minimus* also has a uniquely mediolaterally wider origination surface than any other assessed species, regardless of body mass (Table 2).

Though *Ce*. *minimus* has a proportionally wider pelvis compared to the other sampled taxa (Table S1), the amount of additional origination surface for the m. levator caudae on the postacetabular iliac crest has been previously unrecognized, and likely signals specialized functional use. As males of *Centrocercus* only have ephemeral windows of time to attract mates, the selection pressure for both powerful and prolonged contraction of m. levator caudae has likely been strong (Guan et al., 2022; Vehrencamp et al., 1989; Young et al., 2020). Indeed, previous behavioral studies of *Centrocercus* lekking behavior found that while initial female visitation of males is based primarily on the booming sound they produce, copulation success was associated more closely with the display rate, duration, and repetition of courtship movements (Gibson, 1996). This sustained display consumes massive amounts of energy, to the point that metabolic rate increases by four times relative to when at rest (Vehrencamp et al., 1989). The proportionally large mass and increased origin surface area of this muscle both potentially signal features facilitating prolonged contractile actions of the tail.

The m. lateralis caudae of *Ce. minimus* also has a more expansive origin surface relative to all other assessed species (including those presented here, and in the literature). Additionally, it is second only to *Falco* in proportional mass (Figure 3). Like many other species (*T. furcata*, Ca. *plancus*, *Mer. serrator*, *A. herodias*, *B. jamaicensis*, and *Co*. *coturnix*), it originates on the first two free caudals (Lo Coco et al., 2020; Mosto et al., 2018). However, in *Ce*. *minimus*, it also originates on the dorsocaudal surface of the synsacrum and partially on the postacetabular iliac crest (Figure 1a,c). Distally, the insertion surfaces are similar to other phasianids (*Mel. gallopavo*, *Co. coturnix*) and the anatid *Mer. serrator*, in that they attach to the rectricial bulb dorsolaterally (Harvey et al., 1969). In examined non‐galloanseriforms (*A. herodias*, *B. jamaicensis*, *T. furcata*), the m. lateralis caudae insertion is restricted to the lateral surface of the rectricial bulb, with little discernible dorsal component (Lo Coco et al., 2020). This insertion has also been described as ventrolaterally located in some falconiforms (e.g., *Ca. plancus*) (Mosto et al., 2018). This dorsolateral insertion, possibly unique to the galloanseriforms, suggests that the m. lateralis caudae assists the m. levator caudae in elevating (dorsiflexing) the rectricial apparatus in addition to maintaining the lateral spreading of the tail fan (Mayr, 2009). Indeed, both *Ce*. *minimus* and *Mel*. *gallopavo* appear to show a much more well‐developed dorsal component of the insertion surface of this muscle, which suggests its importance in assisting dorsiflexion of the broad, heavy tail fans that characterize these taxa (McRoberts et al., 2020; Young et al., 2020).

The m. depressor caudae in *Ce*. *minimus* distally expands to a much greater degree than other birds –even other galliforms. This distal expansion strongly contrasts with other groups in which this muscle either distally tapers (e.g., *A*. *herodias*, *B. jamaicensis*, *Mel. gallopavo*, *Co. coturnix*), is nearly parallel‐margined (e.g., *Mer*. *serrator*), or is widest at the midpoint (e.g., *T. furcata*, *Mi. chimango*, Ca. *plancus*) (Lo Coco et al., 2020; Mosto et al., 2018). The proportionally wide distal margins of these muscles may aid in greater ventroflexion of the tail—actions utilized in landing or sudden take‐offs during predator evasion (Young et al., 2020).

### 4.2 Implications and possible causes of unique rectricial bulb morphology

The morphology of the rectricial bulb in *Ce*. *minimus* appears to differ markedly from nearly all previously and newly assessed species with the possible exception of *Mel. gallopavo* (Harvey et al., 1969; Lo Coco et al., 2020; Mosto et al., 2018). In *Ce. minimus*, the bulbs are mediolaterally elongate, with a relatively short craniocaudal width, and with the apices oriented laterally. Like *Ce*. *minimus*, the bulb apices in *B. jamaicensis* and *Co. coturnix* are laterally oriented, but are nearly as elongate as they are wide. Similarly, in *A. herodias* and *Mer. serrator*, the bulbs have a low aspect ratio, but the apices are craniolaterally oriented. These observed differences in the morphology of the rectricial bulb are likely attributable to multiple variables. However, we postulate that two possible contributing variables to these disparities may be the function of the tail fan in courtship display, and the number of rectrices and the corresponding calamus morphology. The wide surface area of the rectricial bulb in *Ce. minimus* and *Mel. gallopavo*, suggests a deviated singular use of this muscle and insertion surface, relative to other species that do not engage in erected tail fans during displays. In these galliforms in particular, upon contraction of the mm. levator caudae and lateralis caudae, the tail fan forms a nearly complete circle in the transverse plane (Figure 1a), perhaps in part facilitated by the proportionally mediolaterally wide rectricial bulb. The number of rectrices and their proportional calamus diameters may also affect rectricial bulb morphology (e.g., mediolateral width). *Ardea herodias*, *B. jamaicensis*, and *Co. coturnix*, on average, have 6 rectrices on either side of the tail. *Meleagris gallopavo* has 8, *Mer. serrator* has 9, and *Ce. minimus* has 10 (Craik et al., 2020; Preston & Beane, 2024; Vennesland & Butler, 2020; Young et al., 2020). Morphology of the calami would also affect the total length, surface area, and subsequent mass of the tail fan. However, the relationship between rectricial bulb morphology and metrics of the associated rectrices requires further investigation.

## 5 CONCLUDING REMARKS

For the first time, we present novel data on the unique tail myology in a critically endangered bird that extensively uses the tail during courtship displays. Though bird tail morphology has evolved primarily under the pressures of maintaining lift during flight, directionally guiding the individual while volant, and aiding in landing, a plethora of living species show that this apparatus can be co‐opted for courtship displays under the influences of sexual selection (e.g., phasianids, trochilids, viduids, paradisaeids). Both species of sage‐grouse (*Centrocercus*) are North American exemplars of this. Though our sample size is limited, the findings presented here support an interpretation that the atypical anatomy and size of the tail muscles in *Ce*. *minimus* are associated with their unique display behaviors. Furthermore, we expect similar findings to be present in other birds that extensively use their tails in courtship displays. Previous studies have also suggested associations between skeletomuscular morphologies and atypical behaviors in birds, such as those in the cranial displays of *Cicinnurus* and *Parotia* (Frith et al., 1998), and unique prey acquisition strategies in extant and extinct birds of prey (Clark et al., 2025). Future studies that not only assess unique behaviors, such as courtship displays, but also the underlying musculature which facilitates them, will help us better understand and appreciate avian biology.

## AUTHOR CONTRIBUTIONS

A.D.C. and J.A. designed the research. A.D.C., J.A., and N.W.S., collected and contributed data. A.D.C. and J.A. supervised the study. S.J.C. produced all illustrations. A.D.C., J.A., S.J.C., and N.W.S. reviewed and edited the manuscript; and A.D.C. wrote the paper.

## Supporting information


Table S1.

## Data Availability

The data that support the findings of this available in the Supporting Information of this article.
